# Patient-Level Savings on Generic Drugs Through the Mark Cuban Cost Plus Drug Company

**DOI:** 10.1001/jamahealthforum.2024.1468

**Published:** 2024-06-14

**Authors:** Ramez Kouzy, Molly B. El Alam, Kelsey L. Corrigan, Hussain S. Lalani, Ethan B. Ludmir

**Affiliations:** 1 Division of Radiation Oncology, The University of Texas MD Anderson Cancer Center, Houston; 2 Program on Regulation, Therapeutics, and Law (PORTAL), Division of Pharmacoepidemiology and Pharmacoeconomics, Department of Medicine, Brigham and Women’s Hospital, Harvard Medical School, Boston, Massachusetts; 3 Department of Medicine, Harvard Medical School, Boston, Massachusetts; 4 Department of Gastrointestinal Radiation Oncology, The University of Texas MD Anderson Cancer Center, Houston; 5 Department of Biostatistics, The University of Texas MD Anderson Cancer Center, Houston

## Abstract

This economic evaluation estimates the out-of-pocket cost savings patients could achieve if generic drugs were purchased directly from the Mark Cuban Cost Plus Drug Company rather than using their health insurance.

## Introduction

In 2022, the Mark Cuban Cost Plus Drug Company (MCCPDC) started selling generic prescription drugs through a transparent, online direct-to-consumer pharmacy model. While potential Medicare Part D plan savings achieved by using MCCPDC have been estimated at over $3 billion for a subset of 77 generic drugs, individual patient-level savings have not been quantified, to our knowledge.^1^ We estimated the out-of-pocket cost savings patients could achieve if they purchased drugs directly from MCCPDC instead of using their health insurance.

## Methods

This economic evaluation used the 2019 Medical Expenditure Panel Survey (MEPS), a publicly available, nationally representative survey of individuals and families. We matched generic medicines sold online by MCCPDC in March 2023 to prescriptions filled in MEPS using codes from the National Drug Code Directory, drug name, strength, and quantity. We focused on tablets and capsules in 30- and 90-pill quantities and identified 124 generic drugs for the analysis (eAppendix in Supplement 1).^2^ Race and ethnicity data were not collected because this study focused on the insurance status of individuals rather than their demographic composition. We followed the CHEERS reporting guideline. This study did not require institutional review based on the criteria of the Common Rule (45 CFR §46) because it used publicly accessible, deidentified data.

To account for potential differences in drug costs from 2019 to 2023, we adjusted out-of-pocket costs in MEPS using the drug-specific percentage change in the National Average Drug Acquisition Cost.^3^ We limited out-of-pocket costs for prescriptions for individuals with Medicaid insurance to $8 per fill, consistent with federal regulations. We compared the difference in patients’ out-of-pocket costs in MEPS with MCCPDC prices to estimate possible savings for a 30- or 90-pill supply of each generic drug, stratified by insurance type (Medicare, Medicaid, military, private, or uninsured), including $5 shipping costs for MCCPDC. Data were weighted to account for the complex survey design. Analyses were performed using R software, version 4.3.0.

## Results

This study identified potential out-of-pocket cost savings in 99 696 682 of 843 713 380 weighted prescription pharmacy fills (11.8%) among 124 generic drugs. No cost savings were observed among patients with Medicaid insurance; percentage of filled prescriptions with savings varied by health insurance: Medicare, 5.5% (14 987 248 of 272 381 168 fills); private, 7.1% (14 679 121 of 207 387 159); military, 9.9% (2 378 664 of 3 993 897); and uninsured, 28.9% (5 000 260 of 12 220 292) (Figure). Median (IQR) estimated cost savings per prescription was $4.96 ($1.95-$11.39); savings were highest for uninsured individuals: $6.08 ($1.87-$10.38). Median (IQR) savings per filled prescription was $5.05 ($2.59-$7.66) for military insurance, $4.64 ($1.89-$10.19) for Medicare, and $3.69 ($1.77-$8.99) for private insurance. Among fills with cost savings, 50.3% had savings less than $5, while 28.4% had savings greater than $10. A sample of generic drugs with savings is presented in the Table.

**Figure.**
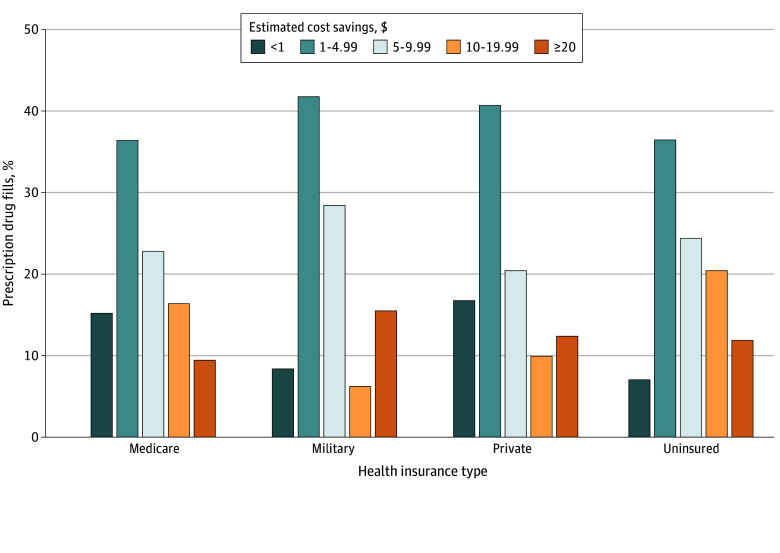
Estimated Cost Savings for Prescription Drugs Among Patients With Any Savings Using Mark Cuban Cost Plus Drug Company Among the 12% of generic prescription fills with savings, we did not observe any cost savings for patients with Medicaid health insurance. We estimated the potential out-of-pocket cost savings for individuals based on their health insurance type in 2019. Of all prescription fills, 5.5% of fills for individuals with Medicare insurance; 9.9%, with military insurance (Veterans Administration or TRICARE); 7.1%, with private insurance; and 28.9%, with no insurance had savings.

**Table.** Out-of-Pocket Cost Savings for Select Generic Prescription Drugs Among Beneficiaries With Any Savings

Drug^a^	Prescriptions (weighted), No.^a^	Proportion of prescriptions with savings, % (95% CI)	Cost savings by insurance type, median (IQR), 2023 $^b^			
			Medicare	Military	Private	Uninsured
Atorvastatin						
30 Pills	35 118 775	7.91 (7.00-8.82)	15.14 (1.50-17.74)	0	4.06 (2.93-4.72)	8.74 (1.86-8.74)
90 Pills	51 836 694	12.59 (11.66-13.51)	4.10 (2.94-6.07)	3.90 (3.90-3.90)	1.89 (0.44-4.57)	6.07 (1.47-7.55)
Amlodipine						
30 Pills	27 397 161	6.31 (5.41-7.22)	3.14 (3.14-8.39)	4.14 (4.14-4.14)	1.30 (1.30-1.30)	6.25 (4.92-6.25)
90 Pills	38 911 361	13.34 (12.27-14.40)	1.97 (1.54-2.94)	5.05 (5.05-5.16)	3.79 (2.94-6.03)	97.24 (0.10-97.24)
Losartan						
30 Pills	16 284 558	18.28 (16.34-20.22)	3.4 (3.4-9.07)	4.58 (4.58-4.58)	3.4 (2.11-3.4)	10.14 (10.14-10.14)
90 Pills	28 837 171	21.81 (20.28-23.34)	5.61 (1.89-6.73)	5.94 (0.36-5.94)	2.35 (1.92-6.70)	6.78 (3.05-21.83)
Fluoxetine						
30 Pills	23 490 172	5.85 (4.80-6.90)	0.55 (0.55-0.55)	0	5.43 (0.55-5.43)	0
90 Pills	11 755 483	10.81 (8.90-12.72)	0.92 (0.25-17.66)	3.03 (3.03-3.03)	5.95 (3.03-17.66)	20.62 (20.62-20.62)
Simvastatin						
30 Pills	10 956 492	13.30 (11.19-15.40)	0.37 (0.37-0.37)	0	4.20 (2.74-4.20)	1.46 (1.46-1.46)
90 Pills	22 689 787	21.09 (19.38-22.79)	3.62 (1.60-8.50)	6.76 (6.76-6.76)	1.80 (0.94-4.42)	1.80 (1.80-1.80)
Rosuvastatin						
30 Pills	8 917 136	14.80 (12.36-17.25)	3.31 (2.51-3.34)	0	2.71 (1.76-3.69)	0.98 (0.98-0.98)
90 Pills	14 990 233	15.24 (13.35-17.13)	8.29 (2.52-17.78)	0	3.12 (1.97-5.37)	0.22 (0.22-0.22)

^a^ The drugs listed were chosen based on the most frequently available prescriptions.

^b^ Savings in 2023 US dollars were determined by subtracting the Mark Cuban Cost Plus Drug Company price (as of March 2023) from the adjusted out-of-pocket payment listed in the Medical Expenditure Panel Survey (MEPS) per prescription. The median (IQR) adjustment in out-of-pocket costs in MEPS was −12% (−34% to 3%) based on changes in the National Average Drug Acquisition Cost between 2019 and 2023.

## Discussion

This economic evaluation found that patients could have spent less on 11.8% of prescriptions filled for 124 generic drugs in 2019 if they had been purchased from MCCPDC instead of using their health insurance. The estimated cost savings was approximately $5 per prescription, including shipping. Savings varied substantially by health insurance type, with uninsured patients achieving the greatest benefit. Our findings are consistent with an analysis of Costco’s direct-to-consumer pharmacy, which found that higher spending occurred in 11% of Medicare Part D claims.^4^ In contrast, an analysis of the 20 most-prescribed generic drugs found that out-of-pocket costs for 20% of prescriptions exceeded prices for Prime members at Amazon Pharmacy.^5^ With generic drugs constituting 90% of all dispensed prescriptions, some patients can benefit from a transparent cost-plus pharmacy pricing model; however, for most, it is less expensive to use their health insurance benefits. Although MCCPDC sells most common generic drugs, only 26% of expensive generic drugs were available in May 2023.^6^

Limitations of our study include its cross-sectional nature. The cost and supply of prescription drugs sold by MCCPDC change frequently. Our analysis was limited to MCCPDC mail-order prescriptions; we did not evaluate the potential cost savings of in-person pickup. Promoting transparent cost-plus pharmacy models, such as MCCPDC, can reduce out-of-pocket costs for a specific subset of patients.

## Notes

Supplement 1. **eAppendix.** Generic Drugs Analyzed

Supplement 2.
Data Sharing Statement
