# Supplementary material for: Patient-Level Savings on Generic Drugs Through the Mark Cuban Cost Plus Drug Company
Source: JAMA Health Forum. 2024 Jun 14;5(6):e241468. doi: 10.1001/jamahealthforum.2024.1468 (PMC11179122; doi:10.1001/jamahealthforum.2024.1468)
Supplement: Supplement 1. — eAppendix. Generic Drugs Analyzed [file jamahealthforum-e241468-s001.pdf]

Supplemental Online Content

Kouzy R, El Alam MB, Corrigan KL, Lalani HS, Ludmir EB. Patient-level savings on generic drugs through the Mark Cuban Cost Plus Drug Company. *JAMA Health Forum*. Published online June 14, 2024. doi:10.1001/jamahealthforum.2024.1468

eAppendix. Generic Drugs Analyzed

This supplemental material has been provided by the authors to give readers additional information about their work.

© 2024 Kouzy R et al. *JAMA Health Forum.*

eAppendix. Generic Drugs Analyzed

1. ACYCLOVIR
2. ALFUZOSIN
3. ALLOPURINOL
4. AMIODARONE
5. AMITRIPTYLINE
6. AMLODIPINE
7. AMOXICILLIN
8. ANASTROZOLE
9. ARIPIPRAZOLE
10. ATENOLOL
11. ATOMOXETINE
12. ATORVASTATIN
13. AZITHROMYCIN
14. BACLOFEN
15. BENAZEPRIL
16. BENZONATATE
17. BUMETANIDE
18. BUPROPION
19. BUSPIRONE
20. CALCITRIOL
21. CARBAMAZEPINE
22. CARVEDILOL
23. CEFDINIR
24. CELECOXIB
25. CEPHALEXIN
26. CETIRIZINE
27. CHLORTHALIDONE
28. CILOSTAZOL
29. CIPROFLOXACIN
30. CLINDAMYCIN
31. CLONIDINE
32. CLOPIDOGREL
33. CYCLOBENZAPRINE
34. CYPROHEPTADINE
35. DIGOXIN
36. DILTIAZEM
37. DIVALPROEX SODIUM
38. DONEPEZIL
39. DOXEPIN
40. ESOMEPRAZOLE
41. ESTRADIOL
42. ETODOLAC
43. EZETIMIBE
44. FAMOTIDINE

© 2024 Kouzy R et al. *JAMA Health Forum.*

45. FENOFIBRATE
46. FEXOFENADINE
47. FINASTERIDE
48. FLUOXETINE
49. FOLIC ACID
50. FUROSEMIDE
51. GEMFIBROZIL
52. GLIMEPIRIDE
53. GLIPIZIDE
54. GLYBURIDE
55. HYDRALAZINE
56. HYDROCHLOROTHIAZIDE
57. HYDROXYZINE
58. IBUPROFEN
59. INDOMETHACIN
60. IRBESARTAN
61. LABETALOL
62. LAMOTRIGINE
63. LEFLUNOMIDE
64. LETROZOLE
65. LEVETIRACETAM
66. LEVOFLOXACIN
67. LEVOTHYROXINE
68. LISINOPRIL
69. LOPERAMIDE
70. LORATADINE
71. LOSARTAN
72. LOVASTATIN
73. MECLIZINE
74. MELOXICAM
75. MEMANTINE
76. METFORMIN
77. METHIMAZOLE
78. METHOCARBAMOL
79. METHOTREXATE
80. METOPROLOL
81. METRONIDAZOLE
82. MIDODRINE
83. MINOCYCLINE
84. MIRTAZAPINE
85. NABUMETONE
86. NADOLOL
87. NAPROXEN
88. NIFEDIPINE
89. NORTRIPTYLINE
90. OLANZAPINE

© 2024 Kouzy R et al. *JAMA Health Forum.*

91. ONDANSETRON
92. OXCARBAZEPINE
93. PIOGLITAZONE
94. PRAMIPEXOLE
95. PRAVASTATIN
96. PRAZOSIN
97. PREDNISONE
98. PRIMIDONE
99. PROGESTERONE
100. PROMETHAZINE
101. PROPRANOLOL
102. RALOXIFENE
103. RAMIPRIL
104. RISPERIDONE
105. ROPINIROLE
106. ROSUVASTATIN
107. SERTRALINE
108. SIMVASTATIN
109. SOTALOL
110. SPIRONOLACTONE
111. TADALAFIL
112. TAMSULOSIN
113. TELMISARTAN
114. TERAZOSIN
115. TERBINAFINE
116. TIZANIDINE
117. TOPIRAMATE
118. TORSEMIDE
119. TRAZODONE
120. VALACYCLOVIR
121. VALSARTAN
122. VENLAFAXINE
123. VERAPAMIL
124. WARFARIN

© 2024 Kouzy R et al. *JAMA Health Forum.*
