# Supplementary material for: Patient-Level Savings on Generic Drugs Through the Mark Cuban Cost Plus Drug Company
Source: JAMA Health Forum. 2024 Jun 14;5(6):e241468. doi: 10.1001/jamahealthforum.2024.1468 (PMC11179122; doi:10.1001/jamahealthforum.2024.1468)
Supplement: Supplement 2. — Data Sharing Statement [file jamahealthforum-e241468-s002.pdf]

Data Sharing Statement

Kouzy. Patient-Level Savings on Generic Drugs Through the Mark Cuban Cost Plus Drug Company. *JAMA Health Forum*. Published June 14, 2024.
doi:10.1001/jamahealthforum.2024.1468

Data
Data available: Yes
Data types: Data (not involving human participants)
How to access data: Request for data can be made to ebludmir@mdanderson.org
When available: With publication

Supporting Documents
Document types: None

Additional Information
Who can access the data: Researchers with reasonable proposed use of data.
Types of analyses: For any purpose.
Mechanisms of data availability: With investigator support
